# Presentation of Complex Congenital Cardiac Anomalies in a Newborn Pediatric Patient: A Case Report

**DOI:** 10.7759/cureus.58596

**Published:** 2024-04-19

**Authors:** Erika E Lytle, Lynne F Holladay

**Affiliations:** 1 Pediatrics, Edward Via College of Osteopathic Medicine, Monroe, USA; 2 Pediatrics, Willis Knighton Health System, Shreveport, USA

**Keywords:** asd (atrial septal defect), transthoracic echocardiogram, congenital heart defect (chd), ventricular septal defect (vsd), tricuspid atresia

## Abstract

Tricuspid atresia, a critical congenital heart defect (CHD), accounts for approximately 1% of all cases of CHDs. When tricuspid atresia is coupled with numerous other unexpected congenital cardiac anomalies, a patient’s condition becomes more serious and more complex. We present a case that demonstrates the stepwise approach to the holistic treatment of congenital tricuspid atresia in the presence of normally related great vessels, a large ventricular septal defect (VSD), atrial septal defect (ASD), and trivial patent ductus arteriosus (PDA). While expanding upon the implementation of chest X-ray imaging, serial transthoracic echocardiogram (TTE) imaging, and the balloon atrial septostomy (BAS) procedure, we also provide insight into the multidisciplinary team-based approach utilized for this patient’s case. This case illustrates a rare critical CHD coupled with other, more common congenital anomalies, and suggests that with multidisciplinary management and treatment, it is possible the mortality rates associated with this diagnosis could decline.

## Introduction

Congenital heart defects (CHDs) affect approximately 1% of all live births in the United States and range in severity from minor to critical [1]. CHDs present in variable degrees of severity, ranging from minor defects, which may have little to no clinical impact, to critical defects, which may require immediate neonatal intervention [2,3]. In critical CHD cases, medical intervention is typically required immediately after birth, as increased severity is associated with marked morbidity and mortality [2,4,5]. 

One critical CHD is tricuspid atresia, which is defined as the failure of the tricuspid valve to form. In tricuspid atresia, patients are born lacking communication between the right atrium (RA) and right ventricle (RV). The underlying etiology of tricuspid atresia is poorly understood; however, it is known to arise due to a disruption of tricuspid valvulogenesis. Physiological atrioventricular valvulogenesis is a multistep process involving the Wnt/β-catenin, bone morphogenetic protein/transforming growth factor-β, and Ras/extracellular-signal-regulated kinase (ERK) pathways [6]. Due to the variability in pathologic disruption of atrioventricular valvulogenesis, patients diagnosed with tricuspid atresia present differently clinically, depending on the specific subtype or degree of pulmonary blood flow [7]. Tricuspid atresia is categorized under three main types, each with distinct subgroups depicting variations in anatomical characteristics and associated conditions. 

Type I is the most diagnosed, with 70% to 80% of tricuspid atresia patients falling into this category. Type I is characterized by normal anatomy of the great arteries and is further divided into subgroups. Subgroup A has an intact ventricular septum with pulmonary atresia, subgroup B has a small ventricular septal defect (VSD) with pulmonary stenosis or hypoplasia, and subgroup C has a large VSD without pulmonary stenosis.

Type II is less commonly diagnosed, comprising 12% to 25% of tricuspid atresia patients. Type II involves D-transposition of the great arteries and is also further divided into subgroups. Subgroup A has a VSD with pulmonary atresia, subgroup B has a VSD with pulmonary stenosis or hypoplasia, and subgroup C has a VSD without pulmonary stenosis.

Finally, Type III is the least commonly diagnosed form of tricuspid atresia, affecting 3% to 6% of patients. Type III encompasses malposition defects, such as truncus arteriosus, atrioventricular septal defects, and double outlet RV, where the great arteries are L-transposed [8].Even so, tricuspid atresia is relatively rare, as it accounts for approximately 1% of all cases of CHDs [9].

Although advances in cardiovascular medicine and surgery over the past few decades have decreased patient mortality and enabled most patients to reach adulthood, CHDs remain the leading cause of mortality due to birth defects and impose a heavy disease burden worldwide [10]. With an evident need for prenatal, postnatal, and lifelong medical care in the management of CHDs, it is imperative for healthcare providers to adequately understand the variety of cardiac anomalies that may present in patients at birth and discern how to treat patients promptly. Moreover, it is essential for physicians, healthcare workers, and caretakers to not only understand the ongoing management of CHD patients but also recognize how to perform as an interdisciplinary unit, with the shared goal of exemplary patient care.

One rather intriguing case is that of a pediatric patient, born with a myriad of complex cardiac anomalies, necessitating a multitude of postnatal imaging studies and surgeries. Just mere hours after birth, the patient received a transthoracic echocardiogram (TTE), as fetal imaging studies identified congenital tricuspid atresia, normally related great vessels, and a VSD. However, the patient’s TTE revealed several unexpected cardiac anomalies that required immediate intervention and follow-up studies. In understanding this patient’s various cardiac defects and the management of care following each diagnosis, physicians can gain insight into the standards of medical care necessary in such a unique setting of complex congenital cardiac anomalies. With an increasing prevalence of critical CHDs, an ever-innovating scientific community must develop a stepwise, holistic management approach that is currently missing from the literature. This clinical scenario proves relevant to physicians and healthcare teams in exploring definitive steps toward managing numerous CHDs discovered in one patient case and the interprofessional synergism necessary for improving patient outcomes.

## Case presentation

**Figure 1:**
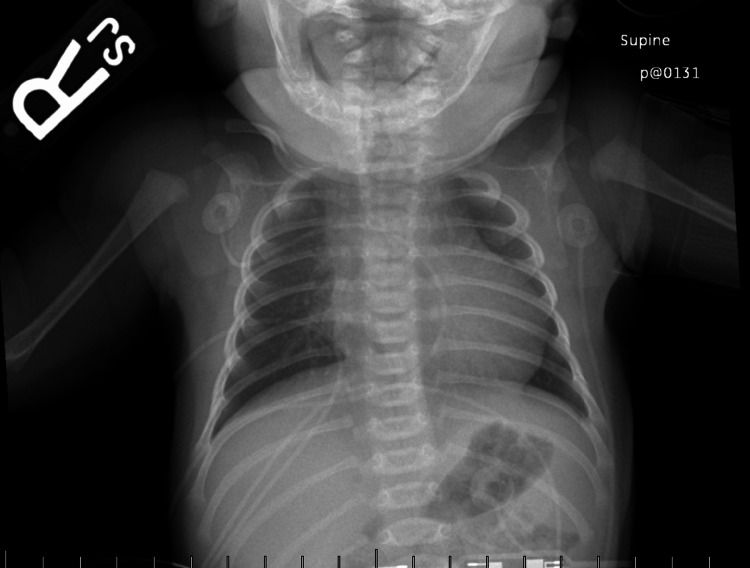
Chest X-ray of the patient upon admission into the CVICU at one day of age. Chest X-ray displays mild cardiomegaly with prominent and granular lung interstitium. CVICU: cardiovascular intensive care unit

A Caucasian newborn female carried to term (39 weeks, 0 days) was delivered vaginally via scheduled induction and presented with a fetal diagnosis of tricuspid atresia, normally related great vessels, and a VSD. Upon delivery, the patient was also noted to have a 4 cm lesion on her sternum consistent with aplasia cutis congenita. The patient was born vigorous. Initial APGAR scores were eight and nine at 1 min and 5 min of life, respectively. Her oxygen saturation was 92% on room air. Her cardiovascular exam revealed a regular rate and rhythm, normal S1 and S2, no S3 or S4, and pansystolic murmur at the lower left sternal border (LLSB). Her respiratory exam revealed symmetric excursion with no retractions or nasal flaring, while her lungs were clear to auscultation with good air movement throughout. Prostaglandin was initiated due to prenatal concern for inadequacy of sufficient pulmonary blood flow. She was transferred to the cardiovascular intensive care unit (CVICU) for further evaluation and management. Upon admission, the chest X-ray displayed mild cardiomegaly with prominent and granular lung interstitium, as seen in Figure 1.

**Figure 2:**
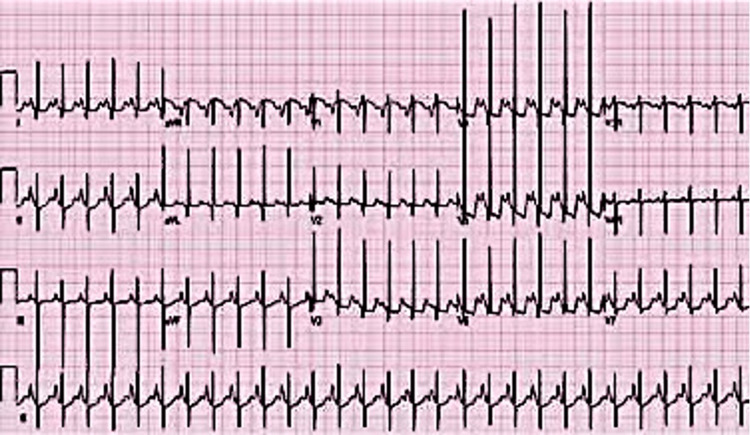
12 lead EKG displaying normal sinus rhythm with abnormal, superiorly oriented mean QRS vector in the frontal plane (-45°, left axis deviation), left ventricular hypertrophy, diminished anterior (R waves in leads V1 and V2), and rightward (S waves in leads V5 and V6) forces. EKG: electrocardiogram

At one day of age, an electrocardiogram (EKG) was completed, which revealed normal sinus rhythm and diminished right ventricular forces with left axis deviation (-45°) and left ventricular hypertrophy, as seen in Figure 2.

A complete TTE was completed on day of life two and revealed plate-like tricuspid atresia, a large VSD, mild pulmonary stenosis, and a severe hypoplastic RV, classifying her as having Type I B tricuspid atresia. There was an aneurysmal tissue noted at the atrial septal defect (ASD) with moderate left to right shunt. The atrial septum was bulging into the left atrium (LA), with the RA noted as normal in size and the LA noted as mildly dilated. The pulmonary valve was thickened and doming but with mild stenosis and no regurgitation, with a peak pulmonary gradient of 25-28 mmHg. The pulmonary valve measured borderline mildly hypoplastic at 0.7 cm with a Z-score of -1.89. The left ventricle (LA) was moderately dilated and hypertrophied with normal systolic function. Finally, the RV function was qualitatively normal with no restriction to flow across the right ventricular outflow tract (RVOT).

**Figure 3:**
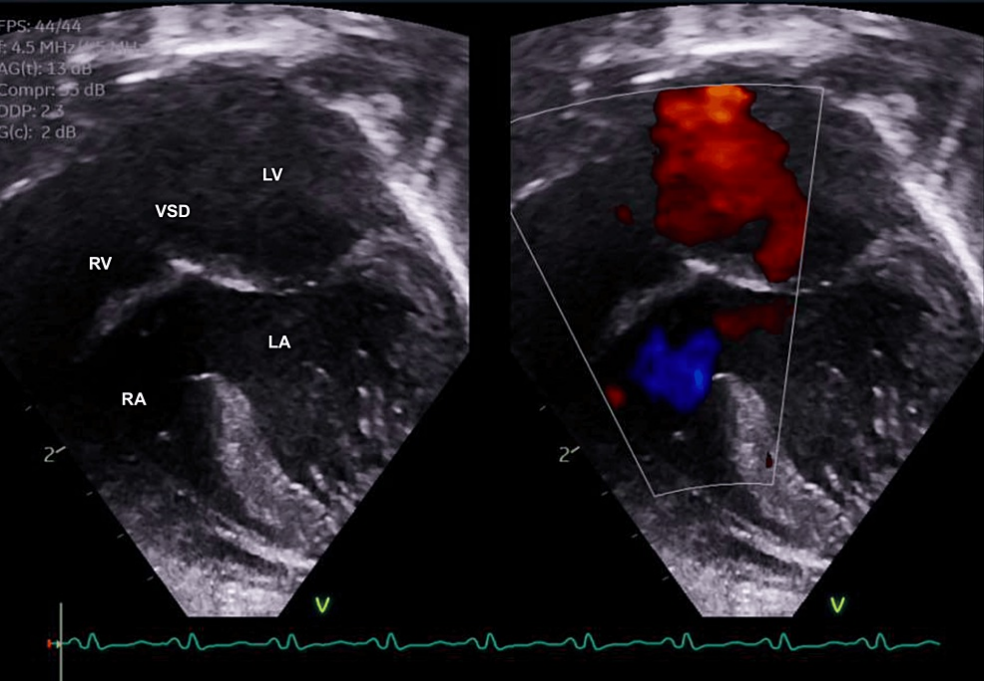
Selected video frames from the patient’s TTE at one day of age showing the patient’s hypoplastic RV paired with a plate-like atretic tricuspid valve and muscular VSD. TTE: transthoracic echocardiogram, RA: right atrium; RV: right ventricle; LA: left atrium; LV: left ventricle; VSD: ventricular septal defect

Other cardiac anomalies became apparent with this imaging modality; more specifically, the TTE displayed the patient also having mild aortic valve insufficiency and a small patent ductus arteriosus (PDA) with left to right shunting, as seen in Figure 3.

With several clearly identified congenital cardiac anomalies, the patient’s healthcare team discussed immediate next steps in her management. Although the patient appeared well clinically, with oxygen saturation levels recorded in the low 90s, there was a concern for possible restrictive ASD as her recent TTE displayed an aneurysmal tissue noted at the ASD. At three days of age, the patient was transferred to the cardiac catheterization laboratory for her echocardiography-assisted balloon atrial septostomy (BAS) procedure. The patient was prepped and draped in the usual sterile fashion and the procedure was guided by TTE. Access was obtained in the right femoral vein with a 6-Fr sheath, using a modified Seldinger technique under ultrasound guidance. A Z-6 balloon-tipped septostomy catheter was introduced into the right femoral vein and advanced across the atrial septum. The balloon was inflated to 2 mL and pulled across the atrial septum and the procedure was repeated with the balloon inflated to 2 mL. The TTE demonstrated an unrestrictive atrial communication and a new atrial defect measuring 6 mm in diameter, with no noted pericardial effusion. The patient was transferred back to the CVICU for monitoring, where she remained on room air. Post-BAS procedure, the patient's oxygen saturation levels dropped and were recorded as being in the high 80s/low 90s, as expected.

At four days of age, the patient’s multidisciplinary healthcare team devised a thorough plan for medical care. She recovered well from her BAS procedure with no complications. Her oxygen saturation ranged from 86-91% on room air, and she was able to tolerate an oral diet. She was discharged home on the day of life six with plans for close outpatient follow-up by her pediatrician and cardiology team. Before discharge, the patient’s parents were educated on the patient’s current condition, instructed on when to call the hospital about her vital signs or overall presentation change, and trained to use the Cardiac High Acuity Monitoring Program (CHAMP) system. In using the CHAMP system, trained family members would closely monitor the patient’s oxygen saturation levels via pulse oximeter, allowing for outpatient data collection and monitoring of the patient's vital signs while not inpatient. Finally, the patient’s noted aplasia cutis congenital was examined by a dermatologist, who determined no acute intervention was required at this time, as the lesion was benign in origin.

The patient was seen by her pediatrician at eight days of age. Upon evaluation by her pediatrician, the patient was found to be well-appearing with minimal cyanosis. The patient's cardiovascular exam revealed a grade III/VI systolic murmur heard beat at the left lower sternal border. Due to the patient’s CHD and the parents' apprehension toward having a neonate diagnosed with CHD, her pediatrician advised the patient’s parents to establish home healthcare to create an individualized home care plan. Accordingly, the home healthcare provider developed a care plan to work with the patient’s parents for 60 days, aiding in the parents’ knowledge and delivery of cardiac disease management, weekly weight checks, instruction on pain management, and management of circulatory status, while ensuring the patient had adequate nutritional status and no signs or symptoms of infection.

The patient was seen by her pediatrician at 15 days of age and again at 29 days of age, where she appeared well-developed and well-nourished on both occasions. The patient achieved a weight gain of 1.74 pounds between appointments, consistent with her mother's report of breastfeeding the patient every three hours. Her heart murmur was still noted on the physical exam.

The patient was advised to follow-up with her pediatrician at two months of age and continue with the plan of care set forth by her cardiology team to receive the Glenn procedure soon, whereby, the surgeon will disconnect the superior vena cava from the heart and connect it to the pulmonary artery, allowing oxygen-poor blood from the upper body to go directly to the lungs [11]. The patient will continue to receive home healthcare, as well as occupational therapy for movement and motor skill activities, and evaluations with a nutritionist on an as-needed basis for weight-gain assessments while on a breast milk diet.

## Discussion

Our patient presented with a myriad of complex congenital cardiac anomalies and required immediate postnatal imaging studies as well as medical and procedural interventions. With fetal imaging demonstrating congenital tricuspid atresia, normally related great vessels, and a VSD, her medical team determined she would require a chest X-ray, EKG, TTE, and delivery in a hospital with quaternary NICU level care soon after birth. With these imaging modalities, the medical team confirmed the patient had numerous cardiac anomalies that necessitated further evaluation and management.

The patient’s management course followed the utilization of a timely chest X-ray, EKG, serial TTE imaging studies, and an echocardiography-assisted BAS. The patient’s chest X-ray displayed mild cardiomegaly with slightly prominent and granular lung interstitium. Notably, patient with increased pulmonary blood flow shows cardiomegaly and prominent pulmonary vasculature in imaging studies, suggesting unrestricted pulmonary blood flow or increased pulmonary blood flow [12]. A TTE soon followed, confirming our patient’s critical CHD, coupled with an ASD, VSD, PDA, hypoplastic RV, and mild aortic valve insufficiency. It was quickly determined that this patient required an echocardiography-assisted BAS, which would create a right-to-left intracardiac shunt; therefore, decompressing the patient's overloaded RV. The patient’s oxygen saturation levels at discharge were recorded as high 80s/low 90s on room air. This finding is expected post-BAS as oxygen-poor blood begins to mix with oxygen-rich blood, causing oxygen levels in the blood to fall, while heart failure improves overall due to the decreased labor on the hypoplastic RV [13].

The BAS was only a temporary palliation; however, the patient is still suffering from a single ventricle defect due to tricuspid atresia. With her TTE displaying mild pulmonary stenosis, the patient’s healthcare team determined the next best approach would be a bi-directional Glenn procedure at four to six months of age. The Glenn procedure will improve blood flow to the lungs, allowing for better oxygenation. During this procedure, the surgeon will disconnect the superior vena cava from the heart and connect it to the pulmonary artery, allowing oxygen-poor blood from the upper body to go directly to the lungs. Consequently, the Glenn procedure allows blood returning from the upper body to flow directly to the lungs and bypass the patient’s single ventricle heart [11]. Even so, there are potential complications associated with this procedure. As Vermaut et al. explain, approximately 30% of patients who receive the Glenn procedure have complications, such as low oxygen level, high blood pressure, a new neurological deficit, pleural effusions, emergency cardiac catheterization, difficulty feeding, the need for reoperation, and cardiac arrest. Furthermore, patients will require at least three days of recovery in the intensive care unit and several more days inpatient for monitoring. The survival rate is approximately 99% immediately following the Glenn procedure, with studies showing an approximate 80% survival rate 11 years after the procedure was initially performed [11].

The paradigm of providing adequate systemic blood flow in the management of tricuspid atresia generally involves three stages. Based on the degree of pulmonary blood flow, pulmonary stenosis, and restriction of the VSD, there are several options available for the initial stage of the three-stage approach. If the patient's LV outflow, aortic valve, and aorta are normal, the patient may only be monitored or receive a pulmonary artery band, pulmonary valve dilation with a stent, PDA stent, or Blalock-Taussig shunt. However, steps two and three are inevitable as the atretic tricuspid valve cannot be utilized. Thus, tricuspid atresia patients will likely undergo the bi-directional Glenn procedure and the Fontan procedure later. Our patient received a septostomy and is scheduled to receive the Glenn procedure and following the literature, will likely receive the Fontan procedure anywhere from two to six years of age. During the Fontan procedure, the surgeon disconnects the inferior vena cava from the posterior aspect of the RA and reconnects it to the pulmonary artery via a conduit [14]. Therefore, in receiving the Fontan procedure, blood will be redirected from the inferior vena cava to the pulmonary artery, allowing blood from the lower body to go directly to the lungs [14]. It is equally important to note that before the bi-directional Glenn and Fontan procedures are performed, patients must undergo a cardiac catheterization to measure pulmonary artery pressure, and consequently assess for hemodynamic and anatomic issues that might complicate or exclude patients from receiving said procedures [15].

Our patient’s condition is particularly rare, as tricuspid atresia accounts for approximately 1% of all cases of CHDs [9]. Moreover, the patient's restrictive atrial septum diagnosed in correspondence with the patient's other heart defects made the understanding of this case and the patient’s expectant management more complex. Additionally, previous studies have shown that large lesions of aplasia cutis congenita are significantly associated with other underlying congenital defects; therefore, it is strongly suggested to investigate for other congenital malformations when discovered [16]. Considering our patient had a 4 cm lesion on her sternum diagnosed as aplasia cutis congenita, an association between this finding and that of other congenital anomalies, such as CHDs, may be considered.

The patient’s mother followed normal prenatal care and the patient’s history was not significant for any known risk factors, making this case even more unique. The etiology of CHD is largely unknown, with only approximately 15% of cases of CHD attributable to a known cause [17]. However, it is well-recognized that several chromosomal aneuploidies are associated with malformation syndromes. As Bouma et al. explain, 8% to 10% of CHD presentations occur in patients with conditions such as Down syndrome, Trisomy 13, Trisomy 18, Turner syndrome, and DiGeorge syndrome, while other risk factors for CHDs include maternal diabetes mellitus, maternal obesity, alcohol use, rubella infection, febrile illnesses, use of certain drugs such as thalidomide and retinoic acid, and exposure to organic solvents [10]. Furthermore, the incidence and mortality of CHDs vary dramatically around the world, as differences in diagnostic capabilities, awareness and accessibility of adequate medical services, and birth defect surveillance systems vary globally [18]. Nevertheless, limited knowledge surrounding the etiologies of CHDs, coupled with the high heterogeneity in CHD epidemics, constitutes major impediments to CHD prevention and early screening.

This clinical case features the necessity of interdisciplinary management to improve patient outcomes, as utilization of the neonatal ICU, pediatric cardiology, pediatric interventional cardiology, pediatric cardiac surgery, nutrition, social work, dermatology, and occupational therapy teams were highlighted. Typical newborn care of neonates with congenital heart disease differs from patient to patient, with more complex CHD diagnoses requiring more commitment from caregivers and medical teams. As March et al. explain in their article outlining parents' perceptions during the transition to home with a neonate having a CHD, monitoring was a stressor for caregivers, with their anxiety exacerbated by feeling as though they were not taught the necessary skills to care for their infants post-discharge. Parents of neonates diagnosed with CHDs overall felt they lacked adequate support once transitioned to home [19]. With tricuspid atresia being a categorically complex CHD, it is anticipated these patients will require increased medical management and treatment as they typically exhibit fatigue, difficulties in feeding, failure to thrive, and recurrent respiratory tract infections [6].

A clinical presentation of critical CHDs is rare and demands not only an extensive understanding of congenital cardiac anomalies but also the incorporation of an interdisciplinary approach. Cohesive healthcare teams have a strong and direct impact on patient outcomes; therefore, the topic of interdisciplinary teamwork in the setting of medical diagnoses remains an imperative discussion within the scientific community. One-year survival for infants with critical CHDs has been improving over time, yet mortality remains high, with just 75% of critical CHD babies living past one year [20]. With multidisciplinary management, it is possible the mortality rates associated with this diagnosis could decline.

## Conclusions

This case demonstrates the stepwise approach to the holistic treatment of a patient with congenital tricuspid atresia in the presence of other CHDs while providing insight into the multidisciplinary team-based approach utilized for this patient. The patient’s multiple heart defects accompanying a rare critical CHD (tricuspid atresia) made this case unique. It is important for healthcare providers to know how to diagnose and treat critical CHDs such as tricuspid atresia, as failure to do so will lead to severe and likely fatal consequences. Future research should focus on the pathophysiology of tricuspid atresia, interstage outcomes, and long-term management of patients with tricuspid atresia, given the growing case literature on the topic.
